# Accelerating HIV Prevention in Cameroon: Factors Associated with Sexual Risk Behaviours and Increased HIV Exposure Among 15–19-Year-Old Adolescent Girls

**DOI:** 10.1007/s10461-025-05023-z

**Published:** 2026-01-19

**Authors:** Ololade Julius Baruwa, Elona Toska, Boladé Hamed Banougnin, Rita Tamambang, Dineo Sekgobela, Pertina Nyamukondiwa, Jane Ferguson, Rachel Yates, Brendan Maughan-Brown

**Affiliations:** 1 Centre for Social Science Research, University of Cape Town, Cape Town, 7701 South Africa; 2 Department of Social Policy and Intervention, University of Oxford, Oxford, United Kingdom; 3 United Nations Population Fund, West and Central Africa Region Office, Dakar, Senegal; 4 Centre for Child and Adolescent Mental Health, College of Medicine, University of Ibadan, Ibadan, Nigeria; 5 Independent Consultant, Geneva, Switzerland; 6 Southern Africa Labour and Development Research Unit, University of Cape Town, Cape Town, 7701 South Africa

**Keywords:** HIV and sexual risk behaviours, Adolescent girls, Inconsistent condom use, Transactional sex, High-risk sex, HIV exposure

## Abstract

Adolescent girls in Cameroon face substantial sexual and reproductive health vulnerabilities that heighten their risk of HIV exposure. Using data from 2,586 adolescent girls aged 15–19 years from the 2017–18 Cameroon Population-based HIV Impact Assessment, we examined factors associated with multiple sexual partnerships, age-disparate sex, inconsistent condom use, transactional sex, and a composite measure of high-risk sex. Overall, 4.31% reported multiple partners, 25.18% engaged in age-disparate sex, 27.06% reported inconsistent condom use, 3.91% engaged in transactional sex, and 19.61% exhibited high-risk sexual behaviours. In multivariable analyses, marriage or cohabitation was strongly associated with age-disparate sex (aOR = 8.57, 95% CI = 5.84–12.56; *p* < 0.001) and inconsistent condom use (aOR = 10.07, 95% CI = 6.98–14.53; *p* < 0.001). Adolescent motherhood was associated with high-risk sex (aOR = 1.71, 95% CI = 1.14–2.55; *p* = 0.011). School non-enrolment was associated with inconsistent condom use (aOR = 1.76, 95% CI = 1.31–2.37; *p* = 0.001). Predicted probabilities showed that unmarried, non-mothers who initiated sex later had the lowest probability of high-risk sex (8%), whereas married adolescent mothers with early sexual debut had the highest (72%). These findings underscore the need for combination HIV prevention programming that prioritises married adolescents, adolescent mothers, out-of-school girls, and those initiating sex early. Delaying sexual debut, preventing adolescent motherhood and child marriage, and strengthening age-appropriate, context-specific interventions may substantially reduce HIV exposure among adolescent girls in Cameroon.

**Supplementary Information:**

The online version contains supplementary material available at 10.1007/s10461-025-05023-z.

## Introduction

 The global fight against HIV/AIDS remains a significant public health challenge, particularly for vulnerable populations such as women and girls. In 2022, women and girls accounted for 46% of new global HIV infections [1]. In sub-Saharan Africa (SSA), adolescent girls and young women (AGYW) aged 15–24 years represent 77% of the global population of people living with HIV, with an estimated 3,100 new infections occurring every week (UNAIDS, 2023). While HIV incidence among adolescent boys and young men (ABYM) has declined by 56% since 2010, reductions among AGYW have been slower, with a 42% decline over the same period [2]. These patterns underscore the need for targeted HIV prevention strategies that address the unique vulnerabilities of adolescent girls.

Cameroon continues to face a generalised HIV epidemic, with an estimated 2.9% of the population living with HIV [3]. AGYW aged 15–24 years remain disproportionately affected, with prevalence rates more than double those of their male peers (1.1% vs. 0.5%) [4]. Although efforts have contributed to a decline in HIV prevalence over the years, adolescent girls continue to face a higher risk of infection compared to their male counterparts [5, 6]. In 2024 alone, approximately 2,474 AGYW aged 15–24 acquired HIV, highlighting the ongoing heightened vulnerability among girls despite overall progress [6].

To understand the factors that shape HIV exposure among adolescent girls, this study applies the Social-Ecological Model [7]. The socio-ecological model posits that health behaviours are influenced by interacting factors across multiple levels: individual, interpersonal, community, and structural. In the context of adolescent girls in Cameroon, individual-level factors include early sexual debut and motherhood status [8, 9]; interpersonal-level factors encompass marital or cohabitation status and household composition [10]; and structural-level factors capture school enrolment, food insecurity, household poverty, and residence in HIV-affected households [11–13]. This framework allows for a holistic examination of how behavioural, social, and structural influences converge to shape sexual risk behaviours and potential HIV exposure.

Evidence from Cameroon and other SSA countries shows that biological, behavioural, and structural factors interact to heighten HIV risks among adolescent girls. Biological susceptibility during adolescence increases vulnerability to HIV acquisition [14–16]. Structural and contextual factors such as gender inequality, poverty, access to healthcare, household structure, and parenting supervision, and limited access to sexual and reproductive health services amplify these risks by constraining adolescents’ ability to protect themselves [15, 17, 18]. Behavioural factors such as multiple sexual partnerships, inconsistent condom use, age-disparate relationships, and transactional sex further elevate HIV acquisition [19–21]. The socio-ecological model captures these interacting levels of influence, highlighting how early marriage, adolescent motherhood, and school dropout intersect with other vulnerabilities to compound HIV exposure risks among adolescent girls in Cameroon.

Although several studies in Cameroon have examined HIV prevalence and behavioural risks among young people, few have focused specifically on sexual risk behaviours among adolescent girls aged 15–19. Most prior research combines data from males and females, masking gender-specific patterns and the intersection of key risk factors [22, 23]. By centering exclusively on adolescent girls, this study addresses a critical gap in the literature by focusing on behavioural patterns that substantially elevate HIV vulnerability. Specifically, it examines multiple sexual partnerships, age-disparate relationships, inconsistent condom use, transactional sex, and a composite indicator of high-risk sex (i.e., engagement in more than one sexual risk behaviour) – all of which are well-established behavioural precursors to HIV exposure and acquisition in SSA [19, 24–26].

In Cameroon, multiple sexual partnerships and low condom use remain significant drivers of HIV exposure. Nearly half of HIV-positive participants in one study reported engaging in multiple partnerships, with inconsistent condom use particularly common among them [23], while youth in Yaoundé and Douala revealed persistently high rates of partner turnover and low condom use, especially in “trusted” regular relationships [22]. Age-disparate and transactional sexual relationships further amplify HIV vulnerability. Relationships with older partners often involve economic and power imbalances, limiting girls’ ability to negotiate condom use [10, 27, 28]. Transactional or economically motivated relationships are similarly associated with higher HIV risk [24, 29]. Collectively, these behavioural patterns, such as multiple partnerships, inconsistent condom use, age-disparate relationships, and transactional sex, represent proximate determinants of HIV exposure, reflecting interlocking economic, social, and gendered inequalities [28, 30].

This study leverages nationally representative data from the 2017–18 Cameroon Population-based HIV Impact Assessment (CamPHIA) to explore how multilevel factors intersect to shape sexual risk behaviours among adolescent girls. Guided by the socio-ecological model and existing literature [7, 31–35], the study examines how marital status, adolescent motherhood, early sexual debut, school enrolment, and food insecurity are associated with sexual risk behaviours and HIV exposure. In addition, the study used predicted probabilities to identify subgroups of adolescent girls most at risk, providing actionable insights to guide targeted, context-specific, and high-impact HIV prevention interventions. Specifically, this study aims to: (1) examine the prevalence of sexual risk behaviours among adolescent girls aged 15–19 in Cameroon; (2) analyse associations between literature-based correlates and sexual risk behaviours; and (3) assess predicted probabilities of high-risk sex. Findings will contribute to the growing body of knowledge on HIV among youth in sub-Saharan Africa and inform future research and programming in the region.

## Methods

This study utilised cross-sectional data from the 2018 CamPHIA survey. CamPHIA is a nationally representative survey designed to provide comprehensive insights into the HIV epidemic in Cameroon [10]. It offers critical data on HIV prevalence, distribution, and determinants, as well as on factors influencing sexual risk behaviours among adults and children.

CamPHIA employed a two-stage stratified cluster sampling design to ensure national representation. First, 489 enumeration areas (clusters) were selected with probability proportional to size across 12 strata representing Cameroon’s geographical regions (with the cities of Douala and Yaoundé as distinct strata). In the second stage, households were randomly selected within each cluster. Within each selected household, all adults aged 15–64 years were eligible to participate in the individual interview and biomarker survey component. The survey was led by the Cameroon Ministry of Public Health in collaboration with the U.S. Centres for Disease Control and Prevention (CDC) and other partners [36]. A full description of the methodology is available in the final report [36]. For this study, we included 2586 adolescent girls aged 15–19 years who were HIV-negative at the time of data collection. No additional inclusion or exclusion criteria were applied.

### Variable Measurement

#### Outcomes: Sexual Risk Behaviours

We assessed five sexual risk behaviours that can increase HIV exposure: multiple sexual partners, age-disparate sex, inconsistent condom use, transactional sex, and high-risk sexual behaviours. *Multiple sexual partnerships* were defined as sexual intercourse with more than one sexual partner in the past 12 months. *Age-disparate sex* refers to having sexual partners who are five years or older in the past 12 months. *Inconsistent condom use* was defined as a self-report of non-use or inconsistent use of condoms with at least one of the three most recent sexual partners in the past 12 months. *Transactional sex* was defined as having sexual intercourse with someone in exchange for material support of any type (including goods and services such as money, gifts, and a place to sleep, among others). *High-risk sexual behaviours*: we created a composite variable called ‘*high-risk sex*,*’* which identified respondents who reported inconsistent condom use along with any of the following: multiple sexual partnerships, age-disparate sex and transactional sex. High-risk sexual behaviours and high-risk sex were used interchangeably in this study.

#### Hypothesised Correlates

Potential vulnerabilities were identified through a literature review of key sexual risk behaviours and the drivers of HIV exposure among AGYW in Cameroon [21, 37, 38]. Hypothesised factors were mapped to the Cameroon PHIA dataset, guiding the final list of correlates tested in this analysis: marital or cohabitation status (married or living as if married), motherhood status, early sexual debut, not enrolled in school, and food insecurity. Some potentially relevant factors identified in the literature, such as parental supervision, exposure to sexual education programs, and psychosocial factors, could not be included because they were not captured in the CamPHIA dataset.

*Married or living as if married* was defined as married or currently living with a partner and was coded as a binary variable. *Motherhood* was defined as a binary variable that identified individuals who had at least one pregnancy that resulted in a live birth. *Early sexual debut* was defined as having had sexual intercourse before the age of 17 years and was coded as a binary variable. *Not enrolled in school* was computed as a binary variable based on adolescent reports of not being enrolled in school at the time of the study. Lastly, *food insecurity* was measured using the variable provided in the Cameroon PHIA dataset, based on the household’s experience in the past month of any member going to sleep hungry. For this analysis, adolescent girls from households reporting at least one instance of hunger were classified as experiencing food insecurity. These hypothesised correlates represent the key exposures of interest in this study, reflecting individual, interpersonal, and structural factors that may influence sexual risk behaviours and potential HIV exposure among adolescent girls in Cameroon.

#### Covariates

Covariates included age, place of residence, household poverty, and HIV-affected households. *Age* was defined as the participant’s current age at the time of the study and was dichotomised into two categories: 15–17 and 18–19 years. *Rural residence* was defined as living in a rural area at the time of the survey. *HIV-affected households* were derived from the number of household members who either tested HIV-positive in the survey blood tests or self-reported a positive HIV status. The variable was coded as ‘No’ (0) for households with no HIV-positive members and ‘Yes’ (1) for households with at least one HIV-positive member. 

*Household poverty* was measured using the *wealth quintile* variable provided directly in the Cameroon PHIA dataset [39]. This variable incorporates indicators of housing quality such as housing quality (including, drinking water source, toilet and cooking facilities, and construction materials for the floor, roof, and walls), asset ownership (e.g., TV, car, motorcycle, land, bank account), presence of livestock or farmland, and household crowding (number of persons per room). Households were categorised into five quintiles, ranging from 1 (poorest) to 5 (richest). Adolescent girls in the first two quintiles (1 and 2) were classified as living in poor households.

#### Data Analysis

This study analysed data from 2586 adolescent girls aged 15–19 in Cameroon. Data checks were conducted to assess completeness and consistency. Variables with missing data were evaluated, and listwise deletion was applied to retain only complete cases, ensuring that estimates were based on fully observed data. The proportion of missing data ranged from 0.0% to 5.61%, which was considered sufficiently low to justify complete case analysis. For the variable with the highest missingness (> 5%), we compared included and excluded cases on key sociodemographic characteristics, finding no significant differences. Therefore, a complete case analysis was deemed appropriate without introducing substantial bias.

A three-stage analytical approach was used to examine factors associated with sexual risk behaviours as proxy measures of increased HIV exposure among adolescent girls. First, we computed univariate analysis, using weighted frequencies for outcome variables, predictors, and covariates. Second, we conducted multivariable analysis. Variables for the multivariable models were selected as priori based on evidence from the literature of their association with sexual risk behaviours among adolescent girls in SSA, including Cameroon [13, 21, 37, 38]. Multivariable logistic regression models were fitted separately for each outcome, incorporating the following hypothesised predictors: motherhood, marital or cohabitation status, early sexual debut, school enrolment, and food insecurity. Covariates included age, rural versus urban residence, household poverty (wealth quintile), and HIV-affected household.

Third, to facilitate interpretation and programmatic relevance, predicted probabilities of high-risk sex were computed for combinations of key vulnerability factors that were significantly associated with high-risk sex outcome in multivariable analysis. This approach allows illustration of the individual and cumulative contribution of factors to the probability of experiencing high-risk sex for adolescent girls with multiple intersecting vulnerabilities (e.g., early sexual debut, married/cohabiting, and motherhood).

All statistical estimates were weighted to ensure the findings are nationally representative. The weighting process accounted for variable probabilities of selection, non-response, and post-stratification adjustments by age, sex, and region, following the CamPHIA 2017–2018 methodology. Given the complex multi-stage survey design, variance estimation required a specialised approach. Standard errors and 95% confidence intervals were computed using the Jackknife repeated replication (JK) method, as outlined in the CamPHIA Technical Report: https://www.togetherforgirls.org/en.

### Ethical Approval

The CamPHIA survey protocol was approved by the Cameroon National Ethics Committee for Research on Human Subjects (CNERSH) and the Institutional Review Boards at the Centres for Disease Control and Prevention (CDC; Atlanta, USA), Columbia University Medical Centre and Westat. All participants provided informed consent prior to participation. For adolescent participants aged 15–19 years, written assent was obtained, along with parental or guardian consent, in accordance with Cameroonian regulations on research involving minors. Participation was voluntary, and confidentiality of all data was strictly maintained.

## Results

### Background Characteristics

We analysed data from 2586 adolescent girls in Cameroon (see Table 1). Multiple sexual partnerships were reported by 4.31% of adolescent girls, while 25.18% engaged in age-disparate sex, and 27.06% reported inconsistent condom use. Additionally, 3.91% reported transactional sex, and 19.61% engaged in high-risk sex.

**Table 1:** Socio-demographic characteristics and outcome of the study population

Variables	Sample size	Percentage (95%CI)	Missing values (%)
*Outcomes*			
Multiple sexual partnerships	104/2544	4.31 (3.40–5.47)	42 (1.62)
Age-disparate sex	654/2441	25.18 (23.10–27.38.10.38)	145 (5.61)
Inconsistent condom use	769/2539	27.06 (24.52–29.77)	47 (1.82)
Transactional sex	109/2526	3.91 (3.11–4.91)	60 (2.32)
High HIV exposure risks	548/2539	19.61 (17.64–21.74)	47 (1.82)
*Hypothesized correlates*			
Married or cohabiting	591/2582	18.88 (16.62–21.37)	4 (0.15)
Motherhood	510/2580	16.79 (14.86–18.92)	6 (0.23)
Early sex	598/2539	20.38 (17.87–22.46)	47 (1.82)
Food insecurity	654/2586	24.15 (21.62–26.87)	0 (0.00)
Not enrolled in school	846/2583	29.83 (27.20–32.60)	3 (0.13)
*Covariates*			
18–19	945/2586	36.14 (34.06–38.27)	0 (0.00)
Rural	1481/2586	49.43 (45.33–53.53)	0 (0.00)
HIV-affected household	202/2586	7.81 (6.55–9.30)	0 (0.00)
Household poverty	1372/2585	42.44 (38.32–46.67)	1 (0.04)

Nineteen percent (18.88%) of the adolescent girls were married or cohabiting. Approximately 20.38% reported sexual activity before the age of 17, and 16.79% had a pregnancy that resulted in a live birth. Nearly a third (29.83%) were not enrolled in school at the time of the survey. Additionally, 24.15% experienced food insecurity. About one-third (36.14%) of the participants were aged 18–19 years, and nearly half (49.43%) resided in rural areas. Furthermore, 7.81% lived in HIV-affected households, and 42.44% came from poor households.

### Multivariable Analysis

**Fig. 1:**
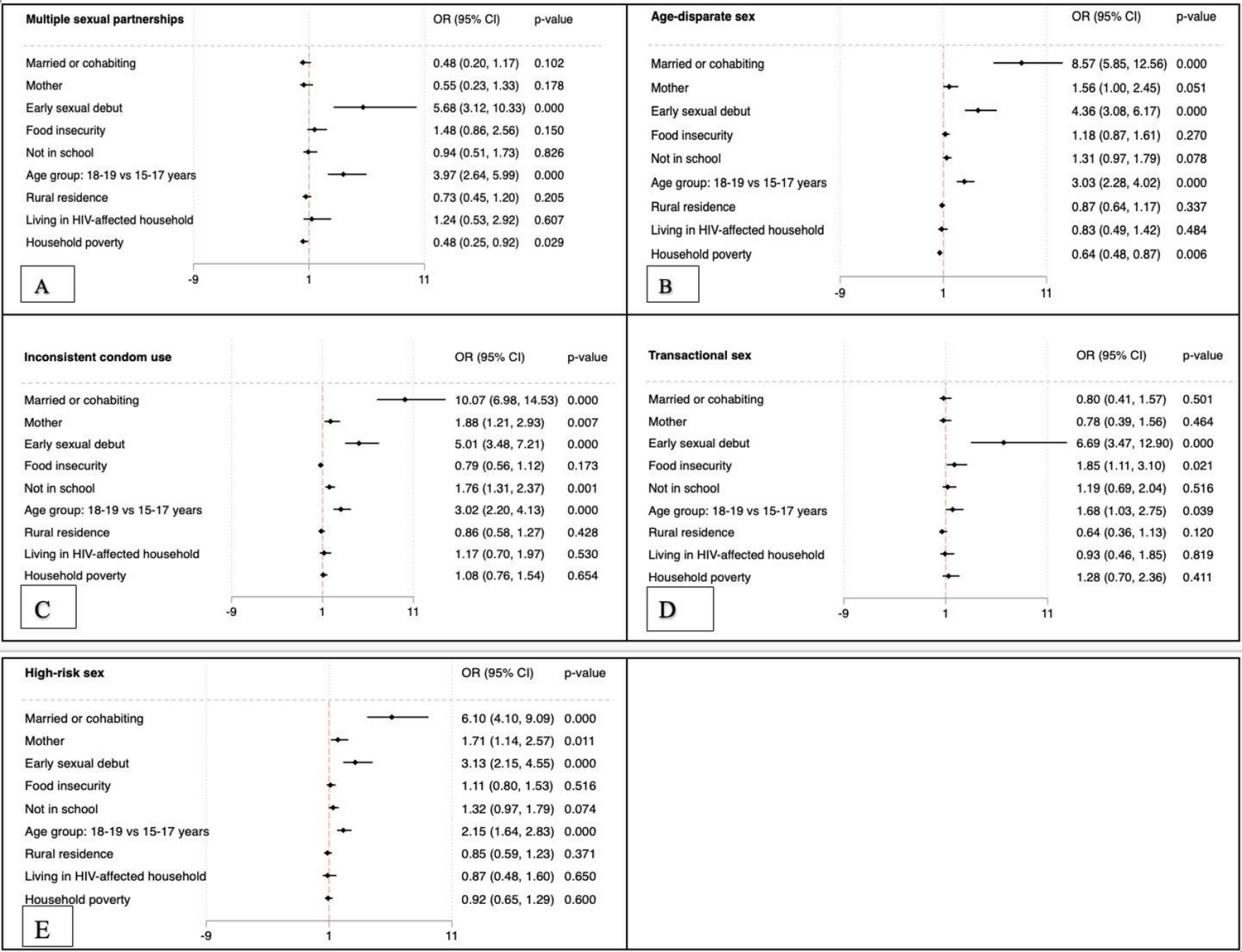
Adjusted odds ratios (ORs) and 95% confidence intervals (CIs) showing factors associated with HIV exposure risks among adolescent girls aged 15–19 years who were HIV-negative in the CamPHIA 2017–2018 survey. **A** Multiple sexual partnerships, **B** Age-disparate sex, **C** Inconsistent condom use, **D** Transactional sex, and **E** High-risk sex

In multivariable analysis (see Fig. 1), early sexual debut, household poverty, and age were significantly associated with multiple sexual partnerships. Adolescents who engaged in sexual activity before the age of 17 were significantly more likely to report multiple sexual partnerships compared to those who delayed sexual initiation (adjusted odds ratio [aOR] = 5.68, 95% CI: 3.12–10.33; *p* < 0.001). Similarly, those aged 18–19 had higher odds of engaging in multiple sexual partnerships compared to their younger counterparts aged 15–17 (aOR = 3.97, 95% CI: 2.64–5.99; *p* < 0.001). In contrast, adolescents from poor households were significantly less likely to engage in multiple sexual partnerships than those from slightly wealthier households (aOR = 0.48, 95% CI: 0.25–0.92; *p* = 0.029).

Factors associated with age-disparate sex among young adolescent girls included being married or cohabiting compared to never-married peers (aOR = 8.57, 95% CI: 5.85–12.56; *p* < 0.001); having sex before the age of 17 (aOR = 4.36, 95% CI: 3.08–6.17; *p* < 0.001); and being 18–19 years old (aOR = 3.03, 95% CI: 2.28–4.02; *p* < 0.001) compared to those aged 15–17 years. Adolescents from poor households were significantly less likely to report age-disparate sexual relationships compared to those from less poor households (aOR = 0.64, 95% CI: 0.48–0.87; *p* = 0.006).

Factors associated with increased inconsistent condom use included being married or cohabiting compared to those who were never married (aOR = 10.07, 95% CI: 6.98–14.53; *p* < 0.001); having given birth (aOR = 1.88, 95% CI: 1.21–2.93; *p* = 0.007); sexual debut before age 17 (aOR = 5.01, 95% CI: 3.48–7.21; *p* < 0.001); and not enrolled in school (aOR = 1.76, 95% CI: 1.31–2.37; *p* < 0.001). Additionally, adolescents aged 18–19 were more likely to use condoms inconsistently than those aged 15–17 (aOR = 3.02, 95% CI: 2.20–4.13; *p* < 0.001).

Adolescent girls who first had sex before the age of 17 were more likely to engage in transactional sex compared to those who first had sex at an older age (aOR = 6.69, 95% CI: 3.47–12.90; *p* < 0.001). The likelihood of transactional sex was also higher among those experiencing food insecurity (aOR = 1.85, 95% CI: 1.11–3.10; *p* = 0.021). Additionally, adolescent girls aged 18–19 were more likely to engage in transactional sex than those aged 15–17 (aOR = 1.68, 95% CI: 1.03–2.75; *p* = 0.039).

Regarding our composite variable of high-risk sex, adolescent mothers were significantly more likely to engage in high-risk sex compared to non-mothers (aOR = 1.71, 95% CI: 1.14–2.57; *p* = 0.011). Those who first had sex before age 17 had higher odds of high-risk sex compared to those who had sex later (aOR = 3.13, 95% CI: 2.15–4.55; *p* < 0.001). Adolescents who were married or cohabiting were also at increased risk compared to their never-married counterparts (aOR = 6.10, 95% CI: 4.10–9.09; *p* < 0.001). Girls aged 18–19 were more likely to report high-risk sex than those aged 15–17 (aOR = 2.15, 95% CI: 1.64–2.83; *p* < 0.001).

### Predicted Probabilities of High-Risk Sex Among Adolescent Girls by Marriage, Motherhood Status, and Early Sexual Debut

**Fig. 2:**
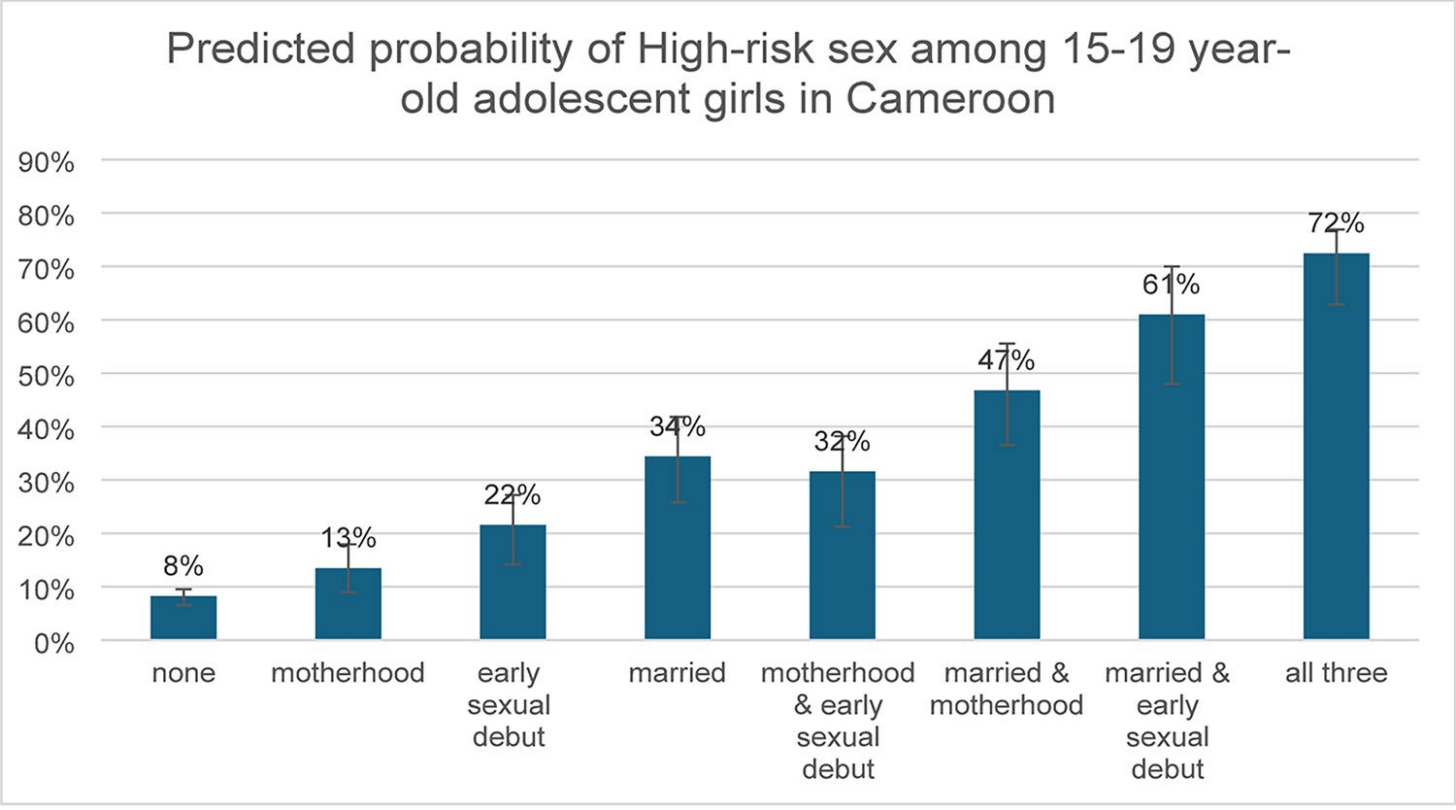
Percentage predicted probabilities of high-risk sex among adolescents by marital status, motherhood, early sexual debut, and school dropout in Cameroon

We computed the predicted probabilities of high-risk sex among adolescent girls by marital status, motherhood, and early sexual debut (see Fig. 2). The findings indicate that girls who were unmarried, not mothers, and delayed their sexual debut beyond 16 years had an 8% predicted probability of engaging in high-risk sex. In contrast, those who were married, mothers, and had an early sexual debut (before age 17) had a substantially higher predicted probability of 72%.

## Discussion

This study examined sexual risk behaviours and HIV exposure among adolescent girls in Cameroon. The findings highlight the key drivers of sexual risk behaviours and HIV exposure, underscoring the intersecting roles of early marriage, motherhood, and early sexual debut in shaping sexual vulnerability, while showing no significant urban–rural differences in sexual risk behaviour outcomes. Predicted probability analysis further illustrated the cumulative nature of these risks; married adolescent mothers who initiated sex early exhibited the highest probability (72%) of engaging in high-risk sexual behaviours, nearly nine times greater than their unmarried, non-mother peers who delayed sexual debut (8%). These findings underscore the intertwined drivers of sexual risk behaviours and increased HIV exposure within social, economic, and gendered contexts in Cameroon.

These results align with the socio-ecological model framework, which explains how structural and relational inequalities shape women’s and girls’ sexual agency. Within the Cameroonian context, patriarchal norms and economic dependency create power asymmetries that constrain adolescent girls’ decision-making in sexual relationships [22, 40]. Consequently, their capacity to negotiate condom use or reject coercive partnerships becomes limited, reinforcing gendered pathways to HIV exposure.

Adolescent marriage emerged as a strong predictor of multiple sexual risk behaviours, including inconsistent condom use, age-disparate sex, and high-risk sexual behaviours. Marriage during adolescence is often characterised by pronounced power imbalances and limited autonomy, which diminish girls’ ability to negotiate condom use, particularly in relationships with older or economically dominant partners [10, 38]. Although marriage is traditionally perceived as protective, the findings align with growing evidence in Cameroon and other countries in SSA that early marriage, especially those occurring under economic or social pressure, can expose girls to heightened sexual and reproductive health vulnerabilities [10, 41, 42].

Adolescent motherhood was also associated with a substantially higher likelihood of inconsistent condom use and high-risk sexual behaviours. Consistent with evidence from Cameroon, adolescent mothers face layered disadvantages, including limited access to reproductive health services, economic dependence, and stigma [8, 43]. Many adolescent mothers remain in financially dependent age-disparate relationships to secure support for themselves and their children [44, 45]. This dynamic erodes their sexual autonomy, increasing exposure to transactional or unprotected sex and perpetuating cycles of vulnerabilities that may expose them to HIV.

Early Sexual Debut was another significant correlate of sexual risk behaviours and HIV exposure, with those who started having sex before 17 years more likely to engage in multiple sexual partnerships, inconsistent condom use, and transactional or age-disparate sex. This corroborates evidence that early sexual debut marks the start of sustained sexual vulnerability [9, 46]. In Cameroon, early sexual debut is often driven by poverty, weak parental supervision, and limited access to sexuality education [13]. Within such contexts, adolescent girls lack the negotiation skills and self-efficacy to practice safe sex, heightening their risk of HIV exposure [25, 47].

Food insecurity also emerged as a critical driver of transactional sex, reflecting the economic vulnerabilities that compel adolescent girls to exchange sex for money or material support. This finding echoes extensive regional evidence linking food insecurity with risky sexual behaviours among adolescent girls [24, 29, 31, 48]. Although local data are limited in Cameroon, similar patterns have been documented in other SSA countries, where adolescents facing food shortages were significantly more likely to exchange sex for money or goods [24, 31, 48].

School non-enrolment was significantly associated with inconsistent condom use in our study, reinforcing evidence that education is a protective factor against HIV exposure. Being in school promotes access to comprehensive sexuality education, positive peer influences, and stronger communication between adolescents and parents regarding sexual health [11, 49]. Conversely, girls who are out of school often face limited access to sexual health information and services, making them more vulnerable to unsafe practices [50].

Regarding covariates, older adolescents (18–19 years) were more likely to engage in risky sexual behaviours than their younger peers (15–17 years), possibly due to increased autonomy and social exposure [21, 49, 51]. Meanwhile, adolescents from poorer households were less likely to report multiple partnerships or age-disparate relationships compared to wealthier peers, suggesting that restricted social mobility and conservative family norms may offer a degree of protection.

### Policy and Programmatic Implications

The findings of this study have strong implications for adolescent health policy and HIV prevention programming in Cameroon and similar contexts. First, age- and gender-responsive interventions are essential to delay marriage and promote equitable partnerships. Enforcing laws against child marriage and integrating empowerment components into adolescent programs to enhance negotiation skills and self-efficacy [52]. Second, support for adolescent mothers should extend beyond pregnancy prevention to include access to postnatal care, reintegration into education, and economic support programs. Such initiatives can help break intergenerational cycles of poverty and sexual vulnerability [45, 53]. Third, addressing food insecurity through social protection programs, such as school feeding schemes, household grants, or targeted nutritional support, can reduce the economic motivations behind transactional sex [24].

Fourth, educational interventions remain critical. Expanding access to comprehensive sexuality education (CSE) and keeping girls in school are effective strategies to build sexual health literacy, promote autonomy, and reduce HIV exposure [52–54]. Finally, HIV prevention efforts should adopt multisectoral collaboration involving government ministries, NGOs, and community-based organisations to address intersecting vulnerabilities holistically. Aligning these interventions with Cameroon’s HIV Strategic Plan (2021–2026) and SDGs 3 and 5 would enhance coherence and support national and global goals to reduce HIV among adolescent girls.

## Strengths and Limitations

This study has several strengths that enhance the credibility and generalizability of its findings. First, it draws on data from the 2018 CamPHIA, a nationally representative survey with a robust sampling design and high-quality data collection protocols. The use of a large sample of adolescent girls provided sufficient statistical power to detect associations between key vulnerability factors and multiple sexual risk behaviours and increased HIV exposure. The study also used rigorous multivariable methods and applied survey weights to account for the complex design. The inclusion of a composite outcome variable, high-risk sex, further strengthened the analysis by capturing overlapping forms of sexual vulnerability.

However, several limitations should be acknowledged. Missing or incomplete data may have affected the precision of some estimates. The cross-sectional nature of the CamPHIA data limits causal inference and introduces temporal ambiguity. Sensitive behaviours such as condom use and transactional sex relied on self-report, which may be subject to recall or social desirability bias, potentially leading to underestimation. The composite high-risk sex variable may not capture all high-risk profiles. For example, adolescents engaging in multiple or transactional sex with consistent condom use may have been excluded. Finally, some potentially relevant factors, such as sexual coercion, intimate partner violence, or peer influence, were not captured in the dataset.

## Conclusion

This study provides critical insights into the intersecting drivers of sexual risk behaviours and increased HIV exposure among adolescent girls in Cameroon. The findings highlight that adolescent marriage, early motherhood, early sexual debut, school non-enrolment, older adolescent age, and food insecurity were significantly associated with sexual risk behaviours and increased HIV exposure. Married adolescent mothers who initiated sex early exhibited particularly high-risk sex profiles, underscoring the compounded effects of gender inequality, poverty, and limited education on girls’ sexual autonomy.

To mitigate these vulnerabilities, multisectoral, gender-transformative, and age-sensitive interventions are urgently needed. Strategies such as delaying marriage, strengthening CSE in schools, expanding youth-friendly sexual and reproductive health services, and integrating economic support measures (e.g., cash transfers or school-feeding programs) are critical pathways to reducing HIV exposure [24, 52, 53]. Implementation should leverage existing delivery platforms, including schools, youth centres, and community health networks, with strong partnerships among government ministries, NGOs, and local organisations. Importantly, adolescent girls’ participation in program design and monitoring should be prioritised to ensure interventions reflect their lived realities and empower them to make informed choices.

## Supplementary Information

Below is the link to the electronic supplementary material.

Supplementary material 1 (DOCX 13.1 kb)

## Data Availability

The datasets analysed are available upon request at https://www.togetherforgirls.org/en.
