# Supplementary material for: Accelerating HIV Prevention in Cameroon: Factors Associated with Sexual Risk Behaviours and Increased HIV Exposure Among 15–19-Year-Old Adolescent Girls
Source: AIDS Behav. 2026 Jan 19;30(7):1986–95. doi: 10.1007/s10461-025-05023-z (PMC13400698; doi:10.1007/s10461-025-05023-z)
Supplement: Supplementary file 1 — Supplementary material 1 (DOCX 13.1 kb) [file 10461_2025_5023_MOESM1_ESM.docx]

Supplementary Table 1. Percentage predicted probabilities of high-risk sex among adolescents by marital status, motherhood, early sexual debut, and school dropout in Cameroon

|  | Probability (%) | Confidence interval | Variable description |
| --- | --- | --- | --- |
| 1 | 0.08 (8) | 0.07-0.10 | Not married, non-mother, and no sex before 17 years |
| 2 | 0.22 (22) | 0.16-0.29 | Not married, non-mother, but had sex before 17 years |
| 3 | 0.13 (13) | 0.09-0.18 | Not married, no sex before 17 years, but a mother, |
| 4 | 0.32 (32) | 0.25-0.42 | Not married, but a mother and had sex before 17 years |
| 5 | 0.34 (34) | 0.27-0.43 | Non-mother, no sex before 17 years, married |
| 6 | 0.61 (61) | 0.52-0.74 | Non-mother, but married and had sex before 17 years |
| 7 | 0.47 (47) | 0.38-0.57 | No sex before 17 years, but married and a mother |
| 8 | 0.72 (72) | 0.68-0.82 | Married, a mother and had sex before 17 years |
